# The Role of Crowd Support on Home Advantage during COVID-19 Restrictions on Italian Football Competitions. Comparison between 2018–19 and 2020–21 Seasons of the Italian Serie A and Serie B Championships

**DOI:** 10.3390/sports10020017

**Published:** 2022-01-30

**Authors:** Matteo Vandoni, Ottavia E. Ferraro, Alessandro Gatti, Luca Marin, Matteo Giuriato, Dario Silvestri, Nicola Lovecchio, Mariangela V. Puci, Vittoria Carnevale Pellino

**Affiliations:** 1 Laboratory of Adapted Motor Activity (LAMA), Department of Public Health, Experimental Medicine and Forensic Science, University of Pavia, 27100 Pavia, Italy; matteo.vandoni@unipv.it (M.V.); alessandro.gatti08@universitadipavia.it (A.G.); luca.marin@unipv.it (L.M.); 2 Unit of Biostatistics and Clinical Epidemiology, Department of Public Health, Experimental and Forensic Medicine, University of Pavia, 27100 Pavia, Italy; ottavia.ferraro@unipv.it (O.E.F.); mariangela.puci@unipv.it (M.V.P.); 3 Laboratory for Rehabilitation, Medicine and Sport (LARMS), 00133 Rome, Italy; 4 Department of Research, ASOMI College of Sciences, 2080 Marsa, Malta; direttore@asomi-osteopatia.com; 5 Department of Neurosciences, Biomedicine and Movement Sciences, University of Verona, 37129 Verona, Italy; matteo.giuriato@univr.it; 6 Faculty of Physical Culture, Unit of Molecular Biology, Department of Health and Natural Sciences, Gdansk University of Physical Education and Sport, 80070 Gdansk, Poland; 7 Department of Human and Social Science, University of Bergamo, 24147 Bergamo, Italy; nicola.lovecchio@unibg.it; 8 Clinical Epidemiology and Medical Statistics Unit, Department of Medical, Surgical, Experimental Sciences, University of Sassari, 07100 Sassari, Italy; 9 Department of Industrial Engineering, University of Tor Vergata, 00133 Rome, Italy

**Keywords:** home advantage, Italian football, COVID-19 restrictions, crowd influence

## Abstract

The home advantage (HA) affects football competitions, especially due to the presence of crowd support. Even though several studies demonstrated that HA (which is influenced by the crowd) decreased in recent years, the empty stadia caused by COVID-19 restrictions offered unique situations to explore and quantify HA. For this reason, we aimed to assess HA in two seasons of the major Italian Championships. We conducted an observational study with the data from the last three seasons of the Italian football championship A–B series, analyzing a total of 2.964 individual game scores. To quantify the HA, the number of points won at home was calculated as a percentage of the total number of points won, home and away. In every season and for every team classification, HA was found (scored points > 50% in home matches). We reported a difference in HA median score for Serie B. Additionally, a difference was found in Serie A for middle-ranking HA median scores in the two seasons compared (*p*-value = 0.017), which was similarly found in Serie B (*p*-value = 0.009). The number of penalties was lower in the season with a crowd compared to one without a crowd (*p* = 0.001). The HA did not disappear in empty stadiums, so there must be other contributing factors. Additionally, we found that the referees were biased by the presence of the crowd in favor of the home teams, and this result could be considered by the football association during referees’ training and formation.

## 1. Introduction

The home advantage (HA) is described as the home team winning 50% or more of the games they have played in [1]. In all professional team sports, is an effect that has been widely studied and was well documented across the years and championships [1,2,3,4,5,6,7], and it occurs in major team sports, especially in football (soccer) [8,9] Several studies reported different potential mechanisms that may offer advantages in favor of the team who play at “home” (herein referred to as the home team and away team) and analyzed many relevant factors for this phenomenon such as the fans’ support [3,10,11]; the playing-field familiarity [3,8,9,12]; the referees’ judgment [7,10,13,14] and the effect of travel on the visitors’ teams [3,6,9]. The latest research conducted by Leite [15] confirmed the HA effects in ten major European football leagues analyzed during the 2015–2016 season; however, Peeters et al. [16] showed a decreasing trend of HA in the last 45 years in English professional football. Several variables interact with each other and greatly influence the increase in HA; however, in accordance with the literature, one of the main variables decisive in the increase in HA is the fans’ support, and its changes between home and away matches [17,18]. For example, it seems that the HA is influenced by a crowd size of up to 20,000; in Australian football, the HA effect was found to only increase with crowd size up to 20,000 visitors [19]. Nevill et al. [10], Peeters and van Ours [16], Pollard and Gomez [20], and van Damme and Baert [21] document the relation between crowd size and home advantage. In recent decades, technological development (match and video analysis, such as a lot of video recordings for use in post-vision interpretation) and the consequent ability to quickly acquire knowledge about team opponents, field structure, and judges made the preparation and adaptation to the competition more accurate [22]. Moreover, recent studies have shown that the crowd seem to bias referees’ decisions, increasing the disciplinary sanction against the away teams and in this way favoring the home team [2,3,13,23]. In fact, some studies reported that red and yellow cards were more often given to the away team compared to the home one [24]. The restrictions imposed to limit the outbreak of the COVID-19 pandemic changed many aspects of everyday life and caused health, social, and economic impairments [25,26,27]. Additionally, sports events were affected, and one of the major and persistent restrictions was the absence of crowds during all individual and team-sport competitions, called door-blinded competitions. These unique situations caused by COVID-19 restrictions allowed us to observe the importance of fans’ support on the increase (or not) in the HA during major football competitions. 

For these reasons, the aim of this study was to investigate the possible influence of fans’ support during the major Italian football competitions (Serie A and Serie B) between the 2018–2019 series (with crowds) and the 2020–2021 series (without crowds) under different aspects related to football matches such as final positions and referees’ decisions.

## 2. Materials and Methods

### 2.1. Study Design

We conducted an observational study with the data from the last seasons of the Italian football championship Serie A and Serie B. We analyzed a total of 2.964 individual game scores obtained from the football data site http://1x2stats.com/it/ITA/Serie-A/classifica-casa-trasferta/ (accessed on 9 September 2021). A total of 1.444 games were played in the 2018–2019 season with the presence of a crowd (760 in Serie A and 684 in Serie B), and 1.520 games were played in the 2020–2021 season without the presence of the crowd (760 in Serie A and 760 in Serie B). 

To quantify the HA, the number of points won at home was calculated as a percentage of the total number of points won considering all the matches [3]. This method has been previously validated by Goumas et al. [28]. The HA was determined during all season matches both with and without a crowd, and subsequently, we provided a difference between the two seasons. Moreover, we investigated the HA between Champions League (UCL)-qualified teams, Europa League (UEL)-qualified teams, and relegated teams. To calculate the HA, we used the percentage of all competition points gained by home teams, that is, the total number of points gained by home teams divided by the total number of points gained by both home and away teams, multiplied by 100. We also analyzed referees’ decisions in Serie A matches such as the mean red and yellow cards, fouls, and penalties per game for home teams and away teams. We finally obtained the data of eighty-four Serie A referees from the site https://it.whoscored.com (accessed on 9 September 2021).

### 2.2. Statistical Analysis

Quantitative variables are shown as the mean and standard deviation (SD) or the median and quartile range (IQR) as appropriate, and qualitative ones are shown as a percentage. The comparison between the two seasons was made using the Mann–Whitney non-parametric test. All the results were reported by grouping teams considering the final rank position (UCL and UEL, middle-rank, and relegated). A level of 0.05 was considered statistically significant (*p* < 0.05). All data were analyzed using the statistical package for PC SPSS 20.0. (Lead Tecnologies Inc., Charlotte, NC, USA) and STATA (StataCorp. 2021. Stata Statistical Software: Release 17. College Station, TX, USA: StataCorp LLC).

## 3. Results

Table 1 shows results about the HA in the 18–19 and 20–21 seasons of the Italian Championship A and B series and, in every season, for every category (Serie A and Serie B), and for the final rank classification, we observed an HA > 50%; for the B series, we found a higher median score of HA in the season with a crowd than in that without a crowd: the HA was 62.8% vs. 56.1% (*p* = 0.002), respectively. For team classification, significant differences were only found for the middle-ranking teams for both the Serie A and Serie B series. More precisely, in either case, the median scores were higher in the crowd seasons than in those without the crowd: Serie A median scores were 59.5 vs. 52.9 (*p* = 0.017), and B Series median scores were 64.1 vs. 56.0 (*p* = 0.009). 

Our results show the points scored home and away in the two seasons of the Italian Championship Serie A and Serie B. We also provide results for the final rank classification both in Serie A and Serie B. We only found differences for Serie B; in detail, the points scored were lower for away matches with a crowd than in ones without (Series B *p* = 0.050; middle-ranking Serie B *p* = 0.026). No significant differences were observed for Serie A and for home matches (data not shown).

Data from referees’ decisions in home matches show that the mean number of penalties was lower in the season with a crowd compared to the one without a crowd (0.10 ± 0.17 vs. 0.21 ± 0.20; *p* = 0.001), while no significant differences were observed in away matches. In addition, we compared home vs. away matches for each season (with/without crowd) and only in the season with a crowd (2018–2019) did we observe a lower mean number of penalties in home matches compared with away ones (0.10 ± 0.17 vs. 0.18 ± 0.20; *p* = 0.02). Finally, the number of yellow and red cards did not change (*p* > 0.05) (data not shown).

## 4. Discussion

The HA in football competitions has been widely studied and is well documented across the years and championships [2,3,4,5,6,12]. Even if HA was influenced by several variables and these variables also interacted with each other, the empty stadia due to the restrictions caused by the COVID-19 pandemic provided a unique opportunity to explore and to quantify the effect of fan support in HA and how it changed across years in the two major Italian championships, Series A and Serie B (2018–2019 with the crowd; 2020–2021 without crowd). Moreover, the empty stadia allowed us to study the influence of the crowd on referees’ disciplinary sanctions; in particular, we focused on the yellow and red card, fouls, and penalty per game differences between home and away teams.

Our main findings showed that HA was also maintained without a crowd; however, crowd impact only affected Serie B matches, while it did not affect, from a statistical point of view, those of Serie A. The results were confirmed by Fischer et al. [29], who stated that crowd support has an impact in teams participating in international tournaments (with contexts exceeding 20,000 spectators). Similar results were also observed when stratifying for team level, and specifically in middle-ranking teams. Previous research on the HA demonstrated that crowds can have a significant influence on team results, favoring a better performance of the home team (through increased confidence and more attacking-type play) and impeding the performance of the away team [12,17,19,30]. Our results confirm previous results with a significant decrease in HA in 20–21 compared to 18–19 (*p* = 0.002) for Serie B; instead, in Serie A, even if there was a change in HA, this was not statistically significant (*p* > 0.05), which could have been caused by the difference in the level and the experience between players of the two championships, in which the lower-level championship players could suffer more from the influence of a crowd. Moreover, in both Serie A and Serie B, in middle-rank teams, we found a decrease in HA in matches without a crowd. This result was not previously reported and could be related to the achievement of permanence in the same championship or to the failure of better results (qualification to the European Championships) that led to less motivation and poor performance. Probably, a partisan home crowd could motivate teams to win at home, creating a supportive climate with songs, encouragement, and emphasizing every played action, but, without the crowd, the importance to win at home or away is not supported or impeded by external influence. Additionally, our results showed differences in penalties per match between home and away teams with the presence of the partisan home crowd, and this difference was annulled without the presence of the crowd, suggesting, as previously reported [31], an active role for the partisan audience to unconsciously bias the referee decisions. Moreover, a significant difference between penalties in the 18–19 season compared to 20–21 was found. In fact, without a partisan crowd, the referee was more inclined to give a penalty against the home team instead of the away team (0.10 and 0.18 in 18–19, 0.21 and 0.18 in 20–21; *p* = 0.001), but with the crowd, there were fewer penalties against home teams per game compared to the away teams, while this difference was not confirmed in the 20–21 season. Conversely, we did not find any significant differences between red cards per game in the two seasons. In contrast to our results, McCarrick et al. [32], analyzing all the European leagues, found that there was an improvement in the away teams’ and a reduction in the home teams’ performances, enough to nullify the HA. Fischer et al. [29] showed that an empty stadium only influenced HA in German Bundesliga 1 and not in minor German leagues. In addition, in previous studies, with the presence of a partisan audience, the home teams received fewer yellow and red cards and fewer fouls compared to the away teams [31,33,34]. Bryson et al. [35] found that the absence of partisan crowds did not influence HA but reported a reduction of one-third in yellow cards for away teams compared to home teams. Additionally, Wunderlich et al. [31] found that the HA is not influenced by the presence of a crowd, but also, he found that the referee was influenced by the partisan crowd. We found that Serie A is not influenced by the absence of the crowd, while there is a reduction in the HA in teams of Serie B. Whereas the referee seems to be significantly affected by the presence of the crowd in favor of the home teams, some studies reported that the away teams act more offensively with a hostile home crowd [5], and so, this could more fouls to be sanctioned. In light of this, without the hostile crowd, we believed that the away teams used a less aggressive way to play and were sanctioned less.

Our study has some limitations. Firstly, we decided not to consider the 2019–2020 season due to the unique situation caused by COVID-19 outbreaks and restrictions. In our opinion, the unknown situation during the halting of the championship and the start in an uncommon year period (summer) could have led to relevant confounders such as concerns of pandemic spread, uncertain contract status, and the motivation or not to return to play. Secondly, despite data being available for the total number of red cards assigned to each team, we did not make a distinction between “direct” red cards and “double yellow cards” red cards. Additionally, we did not report any data on shoots. Wunderlich et al. [31] reported that in the absence of a crowd, home teams were still able to create more shots and shots on target compared to away teams, which suggests that the advantage of home teams can only partly be explained by the presence of spectators. Finally, after the 2019/2020 season, it seems that there have been changes to the sport itself, with a slower pace of play, the introduction of five substitutions, and the advent of a cooling break [36]. In conclusion, as the HA does not disappear in empty stadiums, there must be other contributing factors such as players’ motivation, travel to reach the game destination, and other statistics of the game (such as shots and offensive and defensive actions), which further studies could help to clarify. Even though we did not find any reduction in the HA without a crowd, we found that the referees were biased by the presence of the crowd, which, in a single game, could be advantageous to the home team. This result should be considered by the football association during referees’ training and formation.

## Figures and Tables

**Table 1:** HA median (IQR) in the two Italian football seasons with or without crowd (C) or (WC).

Tournament	HA 18–19 C	HA 20–21 WC	*p*-Value
Serie A	58.3 (4.0–64.8)	54.1 (49.6–58.5)	0.110
Serie B	62.8 (59.6–67.4)	56.1 (52.3–58.4)	0.002 *
Serie A for level			
Champions League (4 teams)	54.4 (52.6–54.8)	53.2 (44.0–56.8)	>0.900
Europa League (3 teams)	60.3 (58.8–62.1)	61.8 (51.3–69.4)	0.827
Middle Ranking (10 teams)	59.5 (54.2–68.4)	52.9 (49.2–57.7)	0.017 *
Relegated (3 teams)	50.0 (36–79.0)	55.0 (39.4–73.9)	0.827
Serie B for level			
Directly Promoted (2 teams)	63.9 (62.7–65.2)	56.3 (56.2–56.5)	0.123
Play-Off (6 teams)	60.0 (55–68.6)	54.8 (51.6–59.7)	0.200
Middle Ranking (8 teams)	64.1 (61.6–67.0)	56.0 (51.4–57.1)	0.009 *
Relegated (3 teams)	61.3 (51.7–68.4)	56.5 (53.1–67.7)	0.827

* Significance set at *p* < 0.05. *p* = *p*-value.

## Data Availability

Data about Serie A and B teams are available from the site http://1x2stats.com/it/ITA/Serie-A/classifica-casa-trasferta/ (accessed on 9 September 2021), while referees’ data are available from the site https://it.whoscored.com (accessed on 9 September 2021).
